# Revisiting the ‘Whys’ and ‘Hows’ of the Warm-Up: Are We Asking the Right Questions?

**DOI:** 10.1007/s40279-023-01908-y

**Published:** 2023-09-02

**Authors:** José Afonso, João Brito, Eduardo Abade, Gonçalo Rendeiro-Pinho, Ivan Baptista, Pedro Figueiredo, Fábio Yuzo Nakamura

**Affiliations:** 1 Centre of Research, Education, Innovation, and Intervention in Sport (CIFI2D), Faculty of Sport, University of Porto, Porto, Portugal; 2 Portugal Football School, Portuguese Football Federation, Oeiras, Portugal; 3 Research Center in Sports Sciences, Health Sciences and Human Development (CIDESD), University of Maia, Maia, Portugal; 4 Faculty of Human Kinetics, University of Lisbon, Lisbon, Portugal; 5 Department of Computer Science, Faculty of Science and Technology, UiT the Arctic University of Norway, Tromsø, Norway; 6 Physical Education Department, College of Education, United Arab Emirates University, Al Ain, Abu Dhabi United Arab Emirates; 7 Research Center in Sports Sciences, Health Sciences and Human Development (CIDESD), Vila Real, Portugal

## Abstract

The warm-up is considered beneficial for increasing body temperature, stimulating the neuromuscular system and overall preparing the athletes for the demands of training sessions and competitions. Even when warm-up–derived benefits are slight and transient, they may still benefit preparedness for subsequent efforts. However, sports training and competition performance are highly affected by contextual factors (e.g., how is the opponent acting?), and it is not always clear what should be the preferred warm-up modalities, structure and load for each athlete and context. Further, we propose that the warm-up can also be used as a pedagogical and training moment. The warm-up may serve several different (albeit complementary) goals (e.g., rising body temperature, neuromuscular activation, attentional focus) and be performed under a plethora of different structures, modalities, and loads. The current commentary highlights the warm-up period as an opportunity to teach or improve certain skills or physical capacities, and not only as a preparation for the subsequent efforts. Moreover, the (justified) call for individualized warm-ups would benefit from educating athletes about exploring different warm-up tasks and loads, providing a broad foundation for future individualization of the warm-up and for more active, engaged, and well-informed participation of the athletes in deciding their own warm-up practices.

## Key Points

**Table:** 

The warm-up is usually a means to an end (e.g., preparing for subsequent performance), but its pedagogical and training potential should be acknowledged.
The warm-up may serve multiple complementary goals (e.g., cognitive and neuromuscular readiness).
The acute effects of the warm-up in reducing injury rates are controversial and require further research.
Athletes could benefit from long-term exposure to different structures, modalities, and loads of warm-ups, shaping the background for future individualization of the warm-ups or even self-warm-ups (i.e., unsupervised warm-ups).

## Premise

In sports, the warm-up is a classical topic that has been debated and studied for years, both in terms of why (or whether) it should be performed and how to be best implemented [1–13]. As the body of knowledge grew, the field became pervaded with terms such as prior or pre-exercise [12, 14], pre-activation [10, 15], post-activation potentiation (PAP) [6, 16], post-activation performance enhancement (PAPE) [13, 17, 18], and active versus passive warm-up [19, 20]. Moreover, the heterogeneous warm-up structures, protocols, study designs, samples, and comparators have resulted in a conflicting body of evidence [4, 5, 7, 8].

At this point, it is probably time to discuss and attempt to clarify some key concepts behind the warm-up. This commentary will focus on critically exploring the reasons for warming up and exploring possibilities of how to implement a warm-up. Most presented concepts could probably be applied to the concept of re-warm-up. However, the re-warm-up moment may present specific spatial, temporal, and material constraints (e.g., intervals of official matches, different venues) [2, 11, 21], requiring a more in-depth discussion that falls outside the scope of our current commentary.

## What is the Warm-Up Used For?

The term ‘warm-up’ suggests elevation of the body temperature [5, 7, 8, 22–24], which is perhaps the first thing that comes to mind when discussing the subject. However, elevation of temperature per se is not very useful in guiding the design and structure of a warm-up, since nearly any active protocol can be used effectively as a warm-up, such as dynamic stretching, PAP, and multimodal protocols (e.g., FIFA 11 +) [2, 3, 25]. Even passive means (e.g., thermal clothes) used in isolation or combined with an active warm-up may effectively elevate the body temperature [5, 20, 26]. Therefore, increasing body temperature is an effect that will result from most warm-up protocols. Nevertheless, the specifics of *how* the warm-up is implemented matter (e.g., [2–4, 11, 12]), so other goals should be considered.

A goal of the warm-up might also be to engage in ‘neuromuscular’ activation or potentiation, that is, to better prepare the body for the demands of the training session or competition [5, 7, 9, 20, 27]. We are aware that the term ‘neuromuscular’ might be used loosely and broadly. Still, the idea behind it is that the warm-up should include some features that promote neuromuscular readiness that will be required later in the training session (or competition) [9, 27]. Of note, this feature would suggest that warm-ups should be specific instead of general (i.e., mimicking some demands of the sport) [28–30]. Things are more complex than that, and this topic will be discussed further below.

The warm-up may also act as a tool to improve readiness and mentally prepare the athlete for subsequent tasks [5, 23, 31–34], thereby transitioning the athlete from the ‘outer world’ and daily life to the specificities of the sports context. These benefits of the warm-up are not presented here as more or less relevant than the elevation of body temperature or neuromuscular potentiation, but as complementary. This time window could also be used for team bonding/strengthening team spirit [35] and/or perfecting sport-specific actions or routines [29, 33]. Since most athletes spend time with the warm-up, the activities performed within it should be well thought out and meaningful. Across an entire season, the accumulated volume of all warm-ups likely represents a significant percentage of the whole training volume, especially if using long warm-ups, which may last up to 45 min [21, 36, 37] or more [38, 39]. Therefore, the warm-up may play a relevant role in the overall learning of content and long-term training adaptations [40].

Additionally, the warm-up could help athletes who feel pain or discomfort (e.g., athletes in rehabilitation). In such cases, performing specific exercises as part of a warm-up routine could help alleviate unwanted symptoms. For example, a single bout of isometric exercises may induce analgesia and decrease inhibition in athletes with patellar tendinopathy [41], potentially helping them to feel better prepared to cope with the subsequent training session. However, we acknowledge the potential ethical risks involved in such practices. For example, could such exercise-induced analgesia be conducive to the athlete trying too hard, going too far, with subsequent aggravation of the pre-existing condition (e.g., injury)? Although such a debate would lead us astray from the main goals of this opinion paper, we acknowledge that this potential role for the warm-up should be carefully discussed between all the relevant agents and be implemented consciously.

The goals delineated so far (i.e., the elevation of body temperature, neuromuscular and mental readiness, special preparation for athletes with pathologies or recovering from injuries) can be trimmed down to a simple statement: the warm-up is expected to prepare the athletes for performing in the subsequent training session or competition [1, 5, 6, 9, 14, 17, 22, 25].

There is also the controversial issue of injury prevention. Despite widespread claims that the warm-up is essential for injury prevention (i.e., risk reduction), there is no data to prove this general belief [3]. Reviews that suggest otherwise are based on the chronic effects of applying a warm-up program and not on the acute effects of the warm-up [9, 12]. To demonstrate that a warm-up acutely reduces injury risk, studies would have to randomly divide teams or athletes into groups, one of which would warm-up, and the others would start the main training session without performing a warm-up. Then, acute injury occurrence (i.e., in that specific session) would be registered prospectively. Given the reduced frequency of injuries in sports and the limited capacity to recruit large numbers of athletes for research, such investigation would hardly be tested or replicated properly.

Currently, we know of no studies that have performed these steps. Again, ethical issues potentially arise. If the warm-up is believed to decrease injury risk, and accepting that the physiological arguments involved are reasonable (e.g., elevation of body temperature), is it ethical to propose such experimental designs? We believe so. Firstly, beliefs in sports sciences may be wrong and should be tested. Secondly, it is unclear whether a no warm-up group would constitute a real no warm-up group: by engaging in the main training session, the athletes would be automatically warming up, and perhaps we would be discussing semantics (as was previously discussed, almost anything can ‘warm-up’ the athlete, even passive means). Technically, ‘no warm-up’ only means there are no organized/structured activities preceding the main exercises of the session; however, one should note there will still be a ‘warming up’ during those main exercises.

The absence of evidence that the warm-up acutely reduces injury risk does not mean there is no such effect; it only means there is currently no proof of that effect. However, in science, we contend that the burden of proof should be on the shoulders of the proponents [42–44]. Therefore, claims that a warm-up is relevant for acute injury prevention should wait for evidence from empirical studies.

Finally, although there are some generic goals of the warm-up (e.g., elevating body temperature), the specific goals of each warm-up (e.g., improving ball control in soccer) may vary depending on the aims of the ensuing training session or competition.

## How to Implement a Warm-Up?

There has been a call for warm-up protocols to be designed to benefit the potentiation of subsequent performance (e.g., PAP and PAPE) [13, 17, 25, 45]. A separate concept would be ‘priming’ [46–48] (e.g., practice in the morning to boost performance later in that day), but this departs from the concept of warm-up. The broader term ‘warm-up’ may be considered an umbrella term, while terms such as PAP and PAPE have narrower meanings. Each approach presents distinct features, such as operating through different underlying mechanisms and acting at different time windows [5, 25, 45]. However, such discussion is beyond the scope of the current commentary. Here, the focus aims at keeping things simple and practical for the coaches.

Broadly speaking, two major implementation categories may apply: no warm-up (either active or passive) and warm-up. However, no warm-up does not mean absence of warming-up effects during the activities of the main part of the training session (as previously discussed). Counter-intuitively, the warm-up is not always clearly superior to no warm-up in terms of immediate performance enhancement for all assessed variables [19, 49, 50] and, even when the warm-up is superior to no warm-up, the magnitude of the effect of such improvement is sometimes small [8].

However, these comparisons between warm-up and no warm-up should be interpreted with caution because (i) lack of differences in certain assessed variables does not mean there would be no differences in other groups of variables and, indeed, within a single study assessing the effects of a warm-up there may differences in some but not all variables (e.g., [17]); (ii) lack of differences in standardized tests does not imply lack of differences in more multifactorial (and difficult to assess) perspectives of performance or injury risk [51, 52]; (iii) given the interindividual variability in response to any training protocol (not exclusive to the warm-up) [53–55], mean values of the assessments may be averaging out potentially relevant differences [56, 57]; and/or (iv) the studies may simply have lacked statistical power (small samples and/or too many outcomes) [58, 59].

Regardless, when considering how to implement a warm-up, maybe we should at least entertain the possibility of not implementing a warm-up, which could even prepare athletes for real-world scenarios, such as arriving late to the venue, with no to minimum time for the warm-up before a competition. Still, implementing a warm-up may at least have the merit of ‘playing it safe’, and numerous possibilities could be considered. Within the warm-up, different modalities may be considered. Several classifications/taxonomies are possible (e.g., general vs specific [60], neuromuscular vs traditional sport-specific [61], functional inertial vs traditional [62]), and most likely none will be unanimous. Here, we present five broad theme-based categories adapted from Cunha et al. [63] that we believe may be useful in adopting a broad perspective on the topic. Importantly, some tasks may apply to different categories, as they may serve multiple goals.

(i) Generic warm-up (i.e., activities not necessarily related to a specific sport, such as running, skipping, light stretching). Interestingly, certain activities could be considered generic in some contexts and specific in others (e.g., skipping would be considered generic in volleyball but is likely to be considered specific in the context of track and field).^1^ However, those tasks are likely to be implemented differently. For example, in our experience as coaches, we often observed how skipping-like drills in team sports are applied with reduced technical concerns or even under complete absence of supervision, something that would hardly occur in track and field.
(ii) Physical conditioning-based warm-up (e.g., strength development, flexibility training). This can include general physical conditioning (i.e., not necessarily related to the overall sport-specific demands) and/or specific physical conditioning (i.e., aimed to improve sport-specific aspects of performance). Indeed, due to pragmatic reasons related to the scheduling of facilities, athletes may arrive earlier and use pre-training physical conditioning that also operates as a warm-up for the main session. This may fit well with the concept of PAPE and allows court/field-time to be entirely devoted to sport-specific, tactical-technical actions.
(iii) Ludic warm-up (having fun, breaking routine). Again, a ludic warm-up may be completely unrelated to the sport (‘general’) or designed to address some sport-specific demands. In this context, we are not referring to any warm-up that was changed to be more ludic; instead, we are referring to warm-ups whose main goal is to be ludic, with all other goals being secondary. These might be especially appropriate to break the routine, circumvent monotony and/or release ‘tension’ in challenging moments.
(iv) Sport-specific warm-up (i.e., activities that are specific to the sport but not necessarily related with the goals of the main training session or competition). An example would be soccer players engaging in low intensity ball dribbling drills, or basketball players performing 1 vs 1 drills with low-pressure opposition.
(v) Training-session specific warm-up (i.e., not only sport specific but also specific for the ensuing training session). An example would be starting the warm-up with a downgraded version of the first main drill of the session (e.g., a less intense and/or less complex version of the drill).

In our opinion, all these warm-up modalities and possible combinations may have their place in sports, and several reasons may support such decision: (i) there will likely be substantial inter- and intraindividual variability in response^2^ to the warm-up [25, 64], that is, different athletes may respond differently and/or in a different time window to the same warm-up, while the same athlete may require different warm-up tasks and/or loads depending on the day; (ii) while sport-specific and training-session specific warm-up routines are potentially the most time-efficient tasks, they may eventually lead to *excessive* monotony[65],^3^ something that could be investigated through understanding how athletes’ perceptions of the same warm-up change in the long term; (iii) exposing athletes to a greater variety of warm-ups may provide them with greater self-awareness and more options for the future [66]; (iv) in our opinion, athletes should have an active role in modulating their warm-up activities, which benefits from having been previously exposed to different warm-up modalities and loads.

In addition, although the warm-up supposedly improves performance, in most cases performance can only be assessed in proxy, standardized tests (e.g., vertical jumps, linear sprinting, pre-programmed change of direction) (e.g., [67, 68]), and rarely in truly sport-specific tests (i.e., tests that would be specific to a particular sport). For example, what does it mean for an athlete to perform better in the first minutes of a soccer match, and how can we relate that to the actual warm-up effects? Is the athlete playing at a slower pace because the warm-up was insufficient or excessive, or due to strategic reasons and/or other contextual variables unrelated to the warm-up? Is the performance in the initial minutes post-warm-up related to the warm-up itself or is it related to how the opponent team has started the match?

Therefore, athletes’ perceptions and preferences about the warm-up should be considered [26, 32, 69], but again, these may depend on the individual repertoire of experiences. If the athletes have only experienced a very narrow set of warm-ups, they may not have sufficient knowledge or experience to assess whether those warm-ups are ‘optimal’ for them. This rationale further suggests the possibility of including an individualized section (either directed by a professional or self-warm-up), where each athlete can perform activities that are subjectively perceived to be most beneficial to the individual, considering that those strategies may vary from day to day, depending on how the athlete feels. In fact, this call for an individualized warm-up is not novel [5, 64, 66], and is part of a larger need to individualize exercise prescription as a whole [55, 70–72]. However, based on evidence generated through mean values, generalized programs may still be useful as a starting point, such as for beginner athletes or for when coaches are still getting to know their athletes.

For practical reasons, this may sometimes require the athlete to arrive earlier at the training venue and perform the individualized warm-up before the collective warm-up. For example, compromises might have to be established if an athlete has certain preferences regarding the warm-up, but the coach also wants to perform some team-bonding activities and/or group- or team-level drills that require some degree of collective warm-up. Consequently, these practices may also require the coaches to define specific moments for performing individual routines during the warm-up.

## Elevating Performance or Generating Fatigue^4^? A Delicate Balance

The warm-up is expected to improve or potentiate performance (at least acutely). However, the warm-up is usually delivered through active means (i.e., exercise); therefore, the load imposed on the athlete should be considered. This brings about two potentially important effects: (i) if the warm-up is too intense and/or too long, it may generate acute fatigue more than it potentiates performance, potentially decreasing performance in the first minutes after the warm-up [73–75]; and (ii) even when this is not the case, the warm-up may contribute to accumulating fatigue that may compound in the final part of the training session or match. Therefore, the warm-up should often be a balancing act: if too little, it may not improve performance; if too much, it may generate excessive fatigue [25, 73]. As such, coaches should think carefully about the most appropriate warm-up duration, intensity, and sequence/order.

## Additional Considerations for Warming Up Before Competitions

The warm-up for competitions could still abide by the previously noted features, but meaningful and relevant differences could be highlighted [5]. Preparing to engage in a training session differs from preparing to participate in a competition, especially if it is an official and/or very important competition. While before training sessions, the warm-up can be used to develop certain skills or capacities, before a competition, the warm-up will more likely fall into the category of a mere tool to improve subsequent performance. Here, especially if the stakes are high, perhaps routine may likely be more relevant than the nature and contents of the warm-up. Suppose the warm-up for the competition is standardized (with minor manipulations of intensity and duration depending on factors such as environmental conditions). In that case, the athletes can focus exclusively on the subsequent performance. By practicing their routines, a sense of comfort and preparedness may ensue, but also a recalibration or retuning of fine motor skills [1, 33].

However, not all competitions are the same; different stakes, different contexts, different opponents, different calendars (e.g., congested vs non-congested fixtures), and different accumulated fatigue levels may arise. This may open the door for more diversified pre-competition warm-ups (e.g., tasks, duration, intensity) that respect individual needs. And, if the stakes are low and/or the competition is expected to be easily surpassed, perhaps the pre-competition warm-up can be used as another opportunity to practice, to train (of course, this should be a tenet of many warm-ups for training sessions). Finally, relevant temporal, spatial, environmental and/or material constraints may force pre-competition warm-up to differ from pre-training warm-up.

## Concluding Remarks

The warm-up is an activity that aims to improve subsequent performance without generating excessive fatigue and has an unclear role in acutely preventing injury. However, the warm-up could also be seen as an opportunity to teach or improve certain skills or capacities. Given the substantial inter- and intra-individual variability in response to training stimuli, and the many different goals that a training session (or competition) may have, a one-size-fits-all solution is unlikely to exist. Coaches may consider implementing different warm-up structures, using different tasks and loads, thereby providing the athletes with a larger practical knowledge base, and potentially building the tools for progressively better individualizing warm-up procedures later. Although these suggestions may extend to the re-warm-up, care must be taken due to specific spatial, temporal, and material conditions that may constraint the re-warm-up differently.

## Footnotes

1 Although a generic warm-up may include activities that could be associated with physical conditioning, they are not performed as such. For example, athletes may run around the court, but just for ‘warming up’ and not particularly focused on running technique or running performance.

2 There is a continuum of responsiveness, and each athlete may be at a different point of that continuum. However, whether that location in the continuum is stable in time (intraindividual variability) or for a given context is largely unknown.

3 Although specificity allows for a continuum of variability in stimuli (i.e., specificity does not necessarily entail repeating the same practices), that continuum is narrower than if non-specific stimuli were also considered. While this narrower spectrum may lead to increased long-term monotony, monotony is not inherently bad [65], and what constitutes excessive monotony is likely individual- and context-dependent.

4 Fatigue is a complex term and is used here merely for convenience. For our goals, the term is being used loosely to denote an acute feeling of tiredness.
